# Size Fractionation of Fluorescent Graphene Quantum Dots Using a Cross-Flow Membrane Filtration System

**DOI:** 10.3390/nano8110959

**Published:** 2018-11-21

**Authors:** Sang-Gu Yim, Yong Jin Kim, Ye-Eun Kang, Byung Kee Moon, Eun Sang Jung, Seung Yun Yang

**Affiliations:** 1 Department of Biomaterials Science, Life and Industry Convergence Institute, Pusan National University, Miryang 50463, Korea; sg.yim0425@gmail.com (S.-G.Y.); kang.ye0525@gmail.com (Y.-E.K.); 2 Center for Multidimensional Carbon Materials, Institute of Basic Science, Ulsan National Institute of Science and Technology, Ulsan 44919, Korea; dibykim@gmail.com; 3 Department of Physics, Pukyong National University, Busan 48513, Korea; bkmoon@pknu.ac.kr; 4 Department of Bio Environmental Energy, Life and Industry Convergence Institute, Pusan National University, Miryang 50463, Korea

**Keywords:** graphene quantum dots, membrane filtration, tangential flow filtration, 2D nanomaterials

## Abstract

Graphene quantum dots (GQDs) have received great attention as optical agents because of their low toxicity, stable photoluminescence (PL) in moderate pH solutions, and size-dependent optical properties. Although many synthetic routes have been proposed for producing GQD solutions, the broad size distribution in GQD solutions limits its use as an efficient optical agent. Here, we present a straightforward method for size fractionation of GQDs dispersed in water using a cross-flow filtration system and a track-etched membrane with cylindrical uniform nanopores. The GQD aqueous suspension, which primarily contained blue-emitting GQDs (B-GQDs) and green-emitting GQDs (G-GQDs), was introduced to the membrane in tangential flow and was fractionated with a constant permeate flow of about 800 L m^−2^ h^−1^ bar^−1^. After filtration, we observed a clear blue PL spectrum from the permeate side, which can be attributed to selective permeation of relatively small B-GQDs. The process provided a separation factor (B-GQDs/G-GQDs) of 0.74. In the cross-flow filtration system, size-dependent permeation through cylindrical nanochannels was confirmed by simulation. Our results demonstrate a feasible method facilitating size fractionation of two-dimensional nanostructures using a cross-flow membrane filtration system. Since membrane filtration is simple, cost-effective, and scalable, our approach can be applied to prepare a large amount of size-controlled GQDs required for high performance opto-electronics and bio-imaging applications.

## 1. Introduction

Graphene quantum dots (GQDs) are nano-sized monolayers or few-layer graphene sheets (below 20 nm) with a two-dimensional hexagonal lattice structure [1,2,3]. GQDs exhibit quantum confinement because the size of the system is comparable with the de Broglie wavelength of an electron, which is not present in bulk graphene [2]. As quantum confinement is dependent on the size of the system [4,5,6,7], GQDs generate different photoluminescence (PL) spectra as a function of the size of the GQD; this is well-known from theoretical predictions and experimental results [8,9]. Thus, GQDs have received tremendous attention in nanoscience and nanotechnology [10,11]; they have been used as a key nanomaterial in diverse applications including sensors, energy conversion, and bioanalysis [12,13,14].

Optical imaging agents have played a crucial role for visualizing living cells in biological studies and detecting biological reactions involving bioactive agents like enzymes and antibodies [15]. Recently, GQDs with tunable optical properties have been used in several biological applications [16,17]. GQDs smaller than 10 nanometers easily pass cell membranes and are very stable without photo-bleaching [18]. Moreover, they exhibit better biocompatibility compared with other nano-sized imaging agents [15,19]. These superior properties make GQDs very promising candidates for cell-imaging [20,21] and many other biological applications [22].

GQDs have been synthesized by diverse methods based on top-down or bottom-up approaches [23,24]. Top-down methods involve cutting process graphene sheets into GQDs by hydrothermal method, laser ablation, electrochemical oxidation, or oxygen plasma treatment [23]. In the case of the bottom-up approach, GQDs are synthesized starting from molecular precursors, such as citric acid, glucose, or aromatic hydrocarbons for high yields [24]. These molecules can be converted into GQDs using different chemical or physical routes, including microwave pyrolysis, polymerization, and dehydrogenation. The cage-opening of a fullerene also can be used [8]. However, these synthetic methods inevitably produce GQDs with a broad size distribution. Since the optical properties of two-dimensional nanomaterials including GQDs depends on their size and shape, separation of GQDs according to size is important to expand the application range of this material as an optical agent [25,26].

Additional separation processes have been used to produce uniform GQDs following synthesis. High-speed centrifugation techniques using cascade sedimentation depending on mass are effective for size fractionation of particles (solutes), but there is a fractionation limitation using centrifugation due to small differences in mass between GQDs [27]. Chromatographic separation methods have been used for GQD purification, but this approach is difficult to obtain large quantities for practical uses [28]. Alternatively, a pressure-driven membrane filtration system has been used for GQD separation [29]. Depending on the pore size of the membrane, solute sizes with selective cut-off can be obtained during filtration process [30]. The membrane filtration system can operate in two modes: Dead-end filtration and cross-flow filtration [31]. In dead-end filtration, the feed flow is subjected to parallel permeation (filtrated) flow and the feed solution completely passes the membrane, similar to filtration with a syringe filter. Filtered matter larger than the pore size accumulates on the membrane surface or remains plugged within the membrane [32,33]. However, severe fouling on the membrane surface occurs during filtration of GQD solutions, thereby requiring additional treatment to ensure high purity [12,29]. In addition, surface cleaning of the membrane or membrane replacement is required to recover the permeate rate. Cross-flow filtration, also known as tangential flow filtration, is a filtration technique in which the initial feed solution passes tangentially along the surface of the membrane [34]. A pressure difference across the membrane drives components that are smaller than the pores through the membrane. Components larger than membrane pores are retained and pass along the membrane surface, thus flowing back to the feed reservoir. This filtration mode could minimize membrane fouling and provides a stable flux compared to dead-end filtration [35,36]. For a given membrane filtration system and feed solution, cross-flow velocity is the primary parameter that determines mass transfer through the membrane [37]. Although cross-flow filtration has been successfully applied to sort 1-dimensional carbon nanotubes by their sizes [38], this membrane system has not been investigated for fractionating 2D nanomaterials, including GQDs.

Here, we report a facile method for separating fluorescent GQDs dispersed in water by size based on cross-flow filtration using membranes with uniform pore size. Selective permeation of relatively small GQDs through the membrane occurred for a given pair of cross-flow velocity and pore size values; this result was confirmed by comparing with simulation results. We evaluated the feasibility of the crossflow membrane system for size fractionation of GQDs by characterizing the selectivity and permeability in membrane filtration, and the fouling on membrane surfaces by comparing with a conventional dead-end filtration system.

## 2. Materials and Methods

### 2.1. Preparation of Fluorescent GQD Suspensions

GQD aqueous suspensions primarily containing blue-emitting GQDs (B-GQDs) and green-emitting GQDs (G-GQDs) were prepared with a bottom-up method from organic compounds [39]. Briefly, 20 g of ethanol, 3 g of 70% nitric acid and 1 g of acetylacetone were mixed and heated in a high-pressure reactor of 60 cm^3^. The reaction proceeded while stirring (120 rpm) in the reactor at 250 °C for 240 h. After the reaction, the blackish solution was diluted 5 times with water and heated at 100 °C to remove volatile components. Then, the solution was filtered through an Advantec 5C filter paper and stored at 25 °C.

### 2.2. Membrane Filtration Tests

In dead-end filtration tests, 20 μg/mL of the GQD solution was filtered at 0.1 bar using a stirred cell system (Millipore Co., Burlington, MA, USA), which has an effective filtration area of 4.1 cm^2^. Filtration experiments were conducted using 25 mm-disc track-etched membranes (No. 110603, Whatman, Inc., Maidstone, UK) with uniformly distributed cylindrical nanopores (50 nm diameter) [40]. The membrane was pre-wetted with DI water for 1 day prior to mounting in the stirred cell to allow polymer chains to swell and reach equilibrium [41,42]. Filtration experiments were conducted at a constant stirring speed and temperature (25 °C). The working pressure was adjusted using N_2_ gas and applied to the feed side. The permeate flux was determined using an electronic balance. Before filtering GQDs, DI water was circulated for 20 min in order to obtain a non-fouled state permeable membrane.

**Figure 1:**
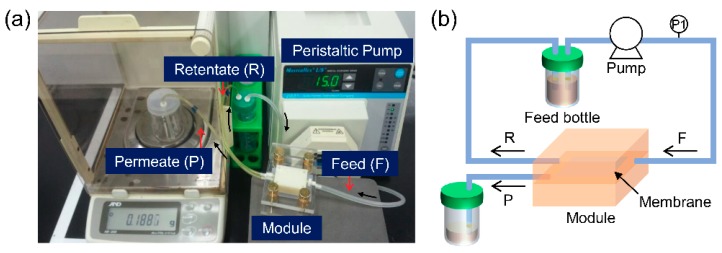
(**a**) Photographic image and (**b**) schematic drawing of the cross-flow filtration system used in this study.

In circulating cross-flow filtration experiments, 20 μg/mL of the GQD feed solution was introduced into a track-etched membrane mounted in a customized module (Figure 1a) and filtered at the same conditions used in the dead-end filtration experiments. The crossflow module had an effective filtration area of 0.5 cm^2^ and was operated with a peristaltic pump (Cole-Parmer, IL, USA) for in order to maintain consistent solution inflow. The feed solution flowed into the upper chamber of the module with 15 mL/min flow rate. The operation pressure was 0.01 bar and was measured with a pressure gauge installed in the feed line. The permeate passing through the track-etched membrane was collected in the lower chamber while the retentate was fed back to the feed solution (Figure 1b). In the cross-flow system, the majority of the feed flow passed tangentially across the membrane surface. The concentrated GQD solutions (retentate) was sampled from the feed bottle and characterized after membrane filtration. The membrane was also pre-wetted with DI water for 1 day prior to mounting in the module. The permeate flux was determined using an electronic balance. DI water was circulated for 2 h in order to obtain a stable flux before filtering the GQDs.

The permeability (flux) of the GQD solutions filtered using the dead-end and cross-flow modes was determined from the filtrated volume per unit area and time (L m^−2^ h^−1^ bar^−1^) as follows (Equation (1)):

(1) $$J\left(L\;m^{-2}\;h^{-1}\;{\mathrm{bar}}^{-1}\right)=\frac{V}{A\cdot t}\times \frac{1}{P}=\frac{m}{A\cdot t\cdot \rho }\times \frac{1}{P}$$

where J is the flux, V is the filtrated volume, A is the effective area of the membrane, t is the operation time, P is the operation pressure, m is the mass, and ρ is the fluid density. To investigate the surface morphology of membranes after GQDs filtration, the membrane surfaces were examined using a field-emission scanning electron microscope (FE-SEM, S-4700, Hitachi, Tokyo, Japan) at 10 kV working voltage. The membranes were coated with platinum using an ion sputter coater (E-1010, Hitachi, Tokyo, Japan) for 40 s.

### 2.3. Simulation of GQDs Transport through Nanochannels

To determine the effective filtration conditions, a transport model for nanoparticles through nanochannels was simulated using computational fluid dynamics (CFD, COMSOL Multiphysics^TM,^ COMSOL INC, Burlington, MA, USA). The Navier–Stokes equation, continuity equation, Brownian force, and drag force were solved in the simulation. The Navier–Stokes equation (Equation (2)) and continuity equation (Equation (3)) for predicting laminar flow describe the motion of a viscous fluid under the assumption that mass is conserved [43]. These equations can be written as

(2) $$0=\nabla \cdot [-pI+\mu (\nabla u+(\nabla u)T)-\frac{2}{3}\mu \left(\nabla \cdot u\right)I]+F$$

and

(3) ∇·(ρu)=0

where p is the fluid pressure, I is the unity tensor, u is the fluid velocity, μ is the fluid dynamic viscosity, and T indicates a transpose operation. The Brownian force (Equation (4)) and drag force (Equation (5)) for predicting particle motion describe the random motion of particles suspended in a fluid and a force acting opposite to the relative motion of any object moving relative to the surrounding fluid [44,45]. These equations can be written as

(4) $$F=\zeta \sqrt{\frac{12\pi k_{B}\mu Tr_{p}}{\Delta t}}$$

and

(5) $$F=\frac{1}{\tau_{p}}m_{p}\left(u-v\right)$$

where ζ is a random number with zero mean, $k_{B}$ is Boltzman’s constant, $r_{p}$ is the particle radius, Δt is the time step taken by the solver, $\tau_{p}$ is the particle velocity response time, $m_{p}$ is the particle mass, and v is the particle velocity.

The designed space of the filtration chamber in the CFD model was subdivided using the finite element method, which is a numerical technique for finding approximate solutions to boundary value problems for partial differential equations (Figure S1). Many contact points were set in and around the membrane for observing fluid and particle motion in detail.

### 2.4. Characterization of GQDs

UV-Vis absorbance and photoluminescence of GQDs were measured to compare the separation efficiency between the two filtration modes. UV-Vis absorbance in the GQDs dispersed in DI water was measured using a UV/VIS spectrophotometer (Optizen 2120UV, Mecasys, Daejeon, Korea). Absorbance was scanned from 200 nm to 600 nm in 1 nm increments. PL spectra from the GQD solutions were obtained using a fluorescence spectrophotometer (FP-6300, JASCO, Tokyo, Japan). Excitation wavelengths were determined from the UV-Vis measurement results. To determine the filtration selectivity between B-GQDs and G-GQDs, the peaks in the PL spectra were deconvoluted using Origin 8.0. Based on the concentration of GQDs calculated from the fluorescence intensity, the selectivity of the membrane was obtained in terms of the separation factor (α) as expressed in Equation (6):

(6) $$\alpha_{p/f}=\frac{C_{p2}/C_{p1}}{C_{f2}/C_{f1}}$$

where $C_{f1,p1}$ are the concentrate of solutes in the feed and permeate solutions at a first emission wavelength, and $C_{f2,p2}$ are the concentrate of solutes in the feed and permeate solution at a second emission wavelength. Peak deconvolutions were performed using Gaussian components.

## 3. Results and Discussion

**Figure 2:**
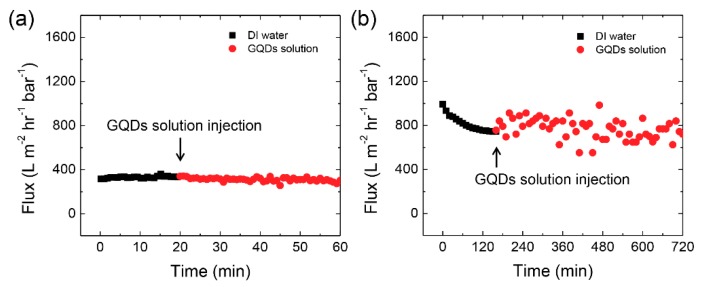
Permeability tests of graphene quantum dot (GQD) solutions with track-etched membranes operated in the two different modes. (**a**) Dead-end filtration and (**b**) cross-flow filtration.

Figure 2 shows the flux of DI water and 20 μg/mL GQDs in aqueous solution through track-etched membranes with uniform cylindrical nanopores using dead-end and cross-flow filtration modes. Dead-end filtration was operated at a constant operation pressure of 0.1 bar, which was controlled with nitrogen gas. The operating pressure in cross-flow filtration was controlled using the rotation speed of a peristaltic pump. In dead-end filtration, the 10 mL of feed solution was completely forced through the membrane for 1 h. The two filtration modes proceeded at a constant permeate flux. The flux was relatively stable in cross-flow filtration for a long period of time (12 h). The permeability of the GQDs solution by cross-flow filtration was two times higher than that operated by dead-end filtration. This flux decrease during dead-end filtration might be due to partial blocking of pores during the initial filtration process.

**Figure 3:**
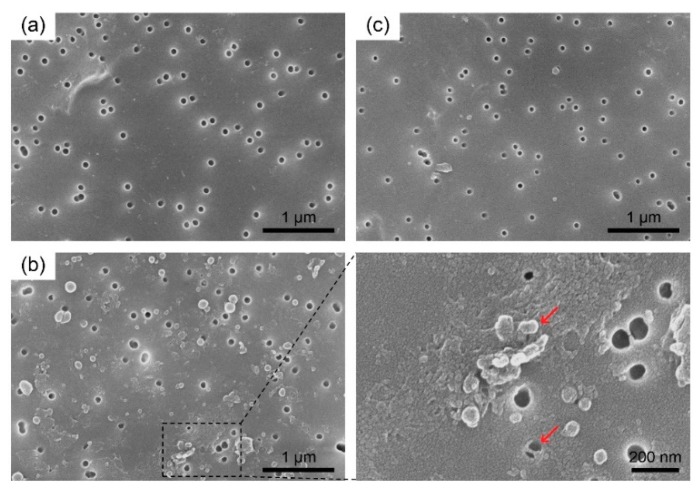
Scanning electron microscope (SEM) images of the top surface of track-etched membranes before and after filtration of GQD solutions in both modes. (**a**) Top surface image of the membrane before filtration. (**b**) Membrane surface at the feed side after dead-end filtration (**left**) and zoomed-in image of the blocked region highlighted by dashed lines (**right**). (**c**) Membrane surface at the feed side after cross-flow filtration.

Figure 3 shows SEM images of the membrane surface at the feed side before and after filtration, respectively.

The track-etched membrane contains uniform cylindrical pores with ~50 nm diameter, 7 um thickness, and surface porosity of ~2% (Figure 3a and Figure S2). After dead-end filtration of the GQD solution (10 mL for 1 h), the pores in the membrane were partially blocked and a cake layer was observed in the specific regions indicated by arrows in Figure 3b. In contrast, cross-flow filtration did not cause significant fouling on the membrane surface, even at large filtration quantity (30 mL) for 12 h (Figure 3c). As anticipated, we confirmed the cross-flow filtration system is more suitable for filtering large amounts while minimizing membrane fouling.

**Figure 4:**
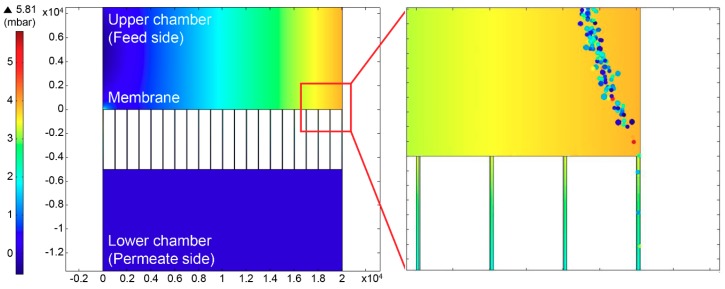
Pressure (**left**) and particle (**right**) trajectories simulated using computational fluid dynamics (CFD) simulations in the cross-flow filtration system.

A simulation was conducted to determine the fluid pressure and particle trajectories between the upper and lower chambers in the cross-flow filtration system. The diameter of model particles was set to 10 nm and 20 nm to estimate the permeation rate of GQDs through the nanochannel. Given the membrane filtration conditions, the cylindrical pore size in the membrane and flow rate were set to 50 nm and 15 mL/min, respectively. The pressure difference between the upper and lower chambers was estimated to be approximately 5 mbar. Considering calculating the simulation time, the number of particles was set to 5000 at a ratio of 1:1. The flow of GQDs was simulated in a laminar condition with a mean velocity of 1.51 × 10^−3^ m/s (Figure 4). As the particles pass through the nanopores, the ratio between 10 nm and 20 nm particles is changed from 0.97 to 1.22. This simulation result suggests that smaller particles have a higher permeate flux in the cross-flow filtration system, thus achieving a selective permeation depending on particle size.

**Figure 5:**
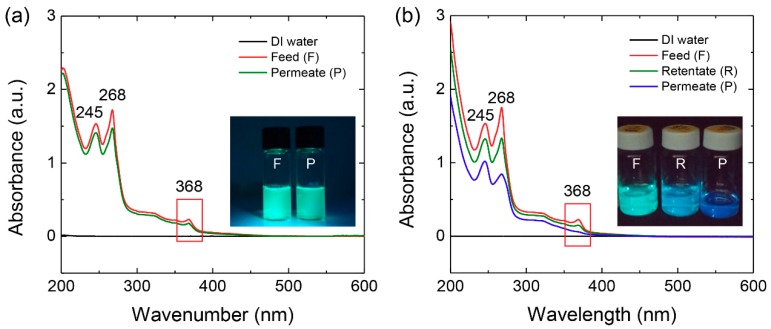
Absorbance spectra of GQD solutions before and after applying the two filtration modes. (**a**) Dead-end filtration and (**b**) cross-flow filtration (Inset: Photographs of the GQD solutions taken under illumination with 365 nm light).

After filtration of the B-GQD/G-GQD mixture, we measured UV-Vis absorbance of permeates obtained from the two filtration modes in order to analyze the separation efficiency. Absorption peaks obtained from the feed and permeate in dead-end filtration were observed at 245, 268, and 368 nm with similar absorption intensities (Figure 5a). The solutions before (feed) and after (permeate) dead-end filtration exhibited a similar blue-green color under UV illumination at 365 nm, indicating filtration did not change the mole ratio of B-GQDs to G-GQDs. In contrast, cross-flow filtration produced a significant difference in the absorption spectra. While the peak position was nearly the same as in the feed and permeate, the absorption peak at 368 nm disappeared in the permeate solution (Figure 5b). A photograph taken of the permeate under UV illumination at 365 nm showed a distinct blue color, highlighting the increased mole fraction of B-GQDs in the B-GQD/G-GQD feed mixture. In the literature, GQDs with diameters ranging from 5 to 10 nm exhibited blue fluorescence under UV illumination at 365 nm [46]. From transmission electron microscopy (TEM) measurements, the average size of GQDs in retentate and permeate sides was estimated to be 5.4 ± 1.1 nm and 2.5 ± 0.3 nm, respectively (Figure S3). This result indicates relatively small GQDs selectively permeate through the nanoporous membrane during cross-flow filtration.

**Figure 6:**
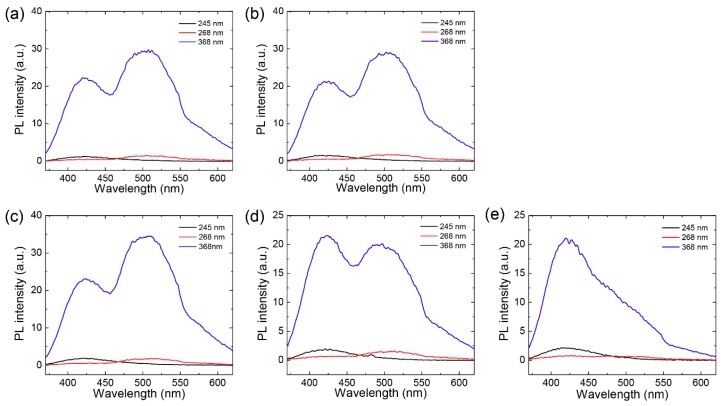
Photoluminescence (PL) spectra from GQD solutions obtained after two filtration modes. (**a**,**b**) PL spectra from (a) feed and (b) permeate solutions in dead-end filtration. (**c**–**e**) PL spectra from (c) feed, (d) retentate, and (e) permeate solutions in cross-flow filtration. The feed solutions used in two filtration modes was prepared by the same synthetic methods of GQDs.

We also measured PL spectra to confirm the separation efficiency. A detailed PL study was conducted using excitation wavelengths of 245, 268 and 368 nm based on the absorbance results to further explore the optical properties of the GQD solutions [47]. Figure 6 shows the PL spectra of GQDs obtained after dead-end and cross-flow filtration, respectively. The GQDs used in this study were strongly excited at 368 nm, resulting in generation of two emission peaks at 420 nm and 520 nm. The feed and permeate solutions obtained from dead-end filtration exhibited similar PL spectra. However, the peak intensity at 520 nm from the permeate solution produced by cross-flow filtration was dramatically reduced with increasing concentration of small B-GQDs in the permeate solution.

After cross-flow filtration, we observed the absorption of GQDs in the interior of circulating tubes in the filtration system and at the membrane module into which the water was introduced. This physical adsorption might be occurred by relatively large GQD with high surface area, resulting in the decrease of PL intensity at 520 nm in the retentate solution (Figure 6d). Since the optical properties of GQDs can be influenced by multiple factors such as chemical functionality and edge-state of GQDs as well as size, more detailed investigation is needed to characterize the size-dependent optical properties of GQDs.

**Figure 7:**
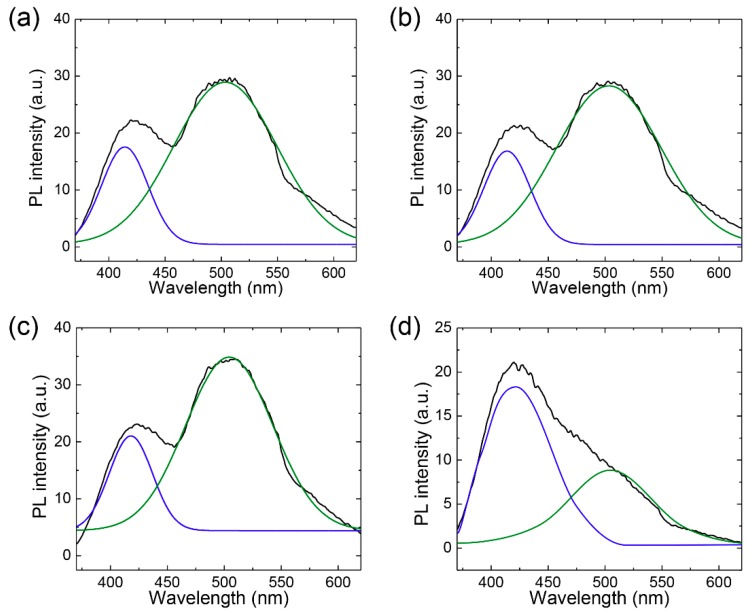
Peak deconvolution in photoluminescence spectra excited at 368 nm. (**a**,**b**) PL spectra from (a) feed and (b) permeate solutions in dead-end filtration. (**c**,**d**) PL spectra from (c) feed and (d) permeate solutions in cross-flow filtration.

For more detailed analysis, we performed deconvolution of the PL spectra at 368 nm excitation to separate the contribution of the two emission peaks (420 nm and 520 nm) and calculate the filtration selectivity (Figure 7). Clear blue PL in the permeate side was attributed to selective permeation of relatively small B-GQDs. We estimate that the selectivity in terms of separation factor (B-GQDs/G-GQDs) between B-GQDs and G-GQDs was 0.74, whereas the separation factor from dead-end filtration was 0.04, meaning B-GQDs were enriched in the permeate side. From these results, we confirmed that cross-flow filtration is a more efficient method than dead-end filtration for separating GQDs by size.

## 4. Conclusions

We first presented a method for separating two-dimensional GQDs using cross-flow membrane filtration with uniform transport nanochannels. A cross-flow membrane filtration system was found to be effective for fractionation of fluorescent GQDs by selective permeation through a membrane, exhibiting 18-fold higher selectivity and 2-fold higher permeability compared to a dead-end filtration system. In addition, cross-flow filtration provides stable flux without significant fouling or flux decline during filtration. Since the membrane filtration process is simple, cost-effective, and scalable, our approach can be used to obtain a large amount of uniform-sized GQDs required for high performance opto-electronic and bio-imaging applications.

## Supplementary Materials

The following are available online at http://www.mdpi.com/2079-4991/8/11/959/s1, Figure S1: Space subdivided by the membrane with nanochannels used in finite element method simulations; Figure S2: SEM images of the cross-sectional image of the membrane before filtration; Figure S3: TEM images of GQDs in (a) retentate and (b) permeate sides obtained after cross-flow filtration. The average size is defined as half of (width (W) + length (L)) of a GQD as shown in the inset of (a).
