# Supplementary material for: Size Fractionation of Fluorescent Graphene Quantum Dots Using a Cross-Flow Membrane Filtration System
Source: Nanomaterials (Basel). 2018 Nov 21;8(11):959. doi: 10.3390/nano8110959 (PMC6267503; doi:10.3390/nano8110959)
Supplement: Supplementary file 1 [file nanomaterials-08-00959-s001.zip › nanomaterials-387829-SI.pdf]

Nanomaterials 2018, 8, 959; doi:10.3390/nano8110959

Supplementary materials

Size Fractionation of Fluorescent Graphene Quantum Dots using a Cross-flow Membrane Filtration System

Sang-Gu Yim, Yong Jin Kim, Ye-Eun Kang, Byung Kee Moon, Eun Sang Jung* and Seung Yun Yang*

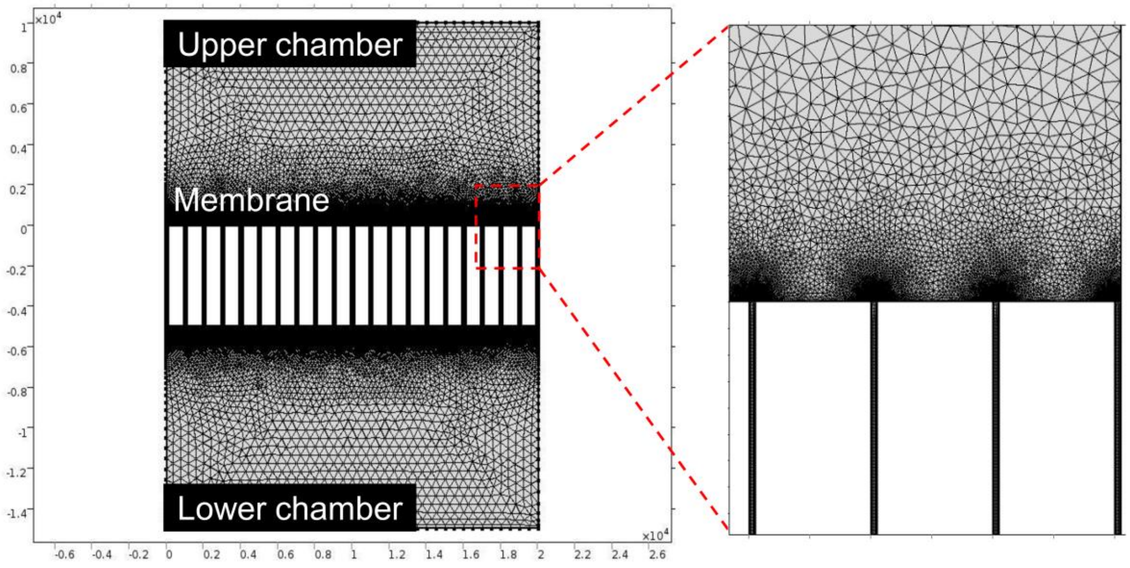

Figure S1. Space subdivided by the membrane with nanochannels used in finite element method simulations.

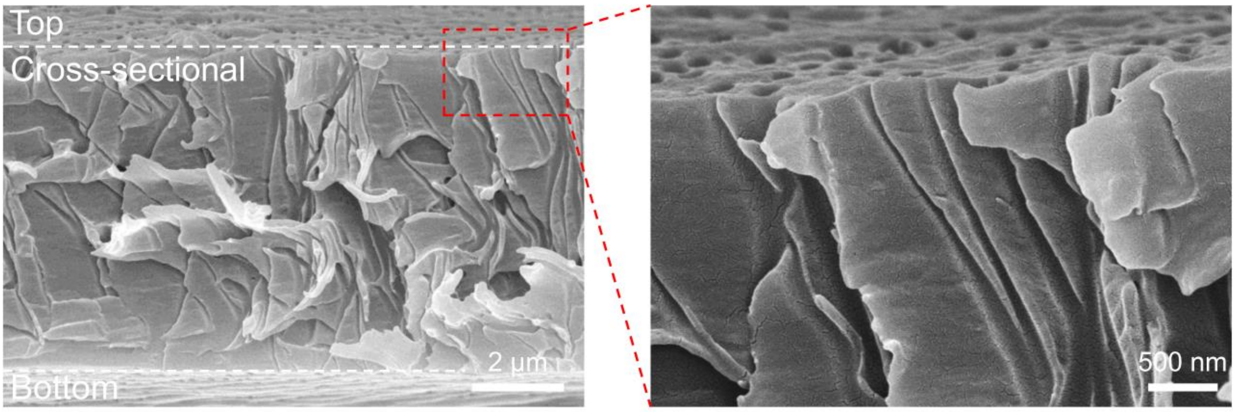

Figure S2. SEM images of the cross-sectional image of the membrane before filtration.

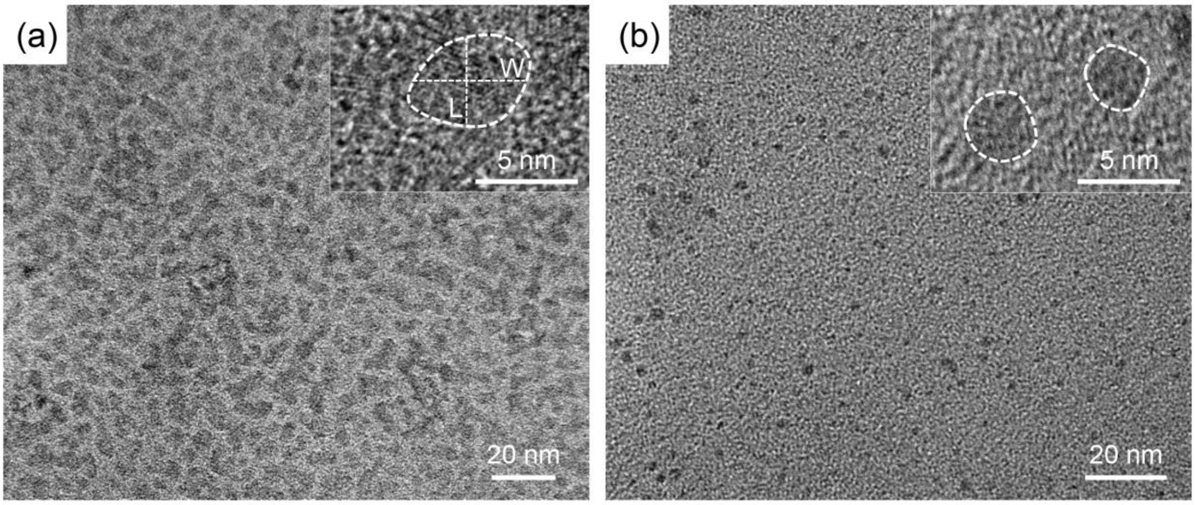

Figure S3. TEM images of GQDs in (a) retentate and (b) permeate sides obtained after cross-flow filtration. The average size is defined as half of (width (W) + length (L)) of a GQD as shown in the inset of (a).
